# A Novel Recyclable Strategy for Extraction of Naproxen Sodium from Environmental Water by Amino-Functionalized Immobilized Ionic Liquid Polymers

**DOI:** 10.3390/molecules30112404

**Published:** 2025-05-30

**Authors:** Hongrui Yang, Ayiguli Maimaiti, Wei Liu, Wenye Deng, Xiaoping Fu, Jing Fan

**Affiliations:** 1 Xinjiang Key Laboratory of New Energy Materials and Green Chemical Engineering, College of Chemical and Environmental Engineering, Xinjiang Institute of Engineering, Urumqi 830023, China; yangyangyang321200@163.com (H.Y.); guzal2006@126.com (A.M.); lwmjp@163.com (W.L.); wenyedeng@sina.com (W.D.); fuxiaoping2020@163.com (X.F.); 2 School of Environment, Key Laboratory of Yellow River and Huai River Water Environment and Pollution Control, Ministry of Education, Henan Key Laboratory for Environmental Pollution Control, Henan Normal University, Xinxiang 453007, China

**Keywords:** amino-functional ionic liquid polymer, naproxen sodium, solid-phase extraction, aqueous environments

## Abstract

Naproxen sodium is an emerging pollutant that may pose potential hazards to human health and the ecological environment. However, developing highly effective adsorbents for the extraction of naproxen sodium from aqueous environments is still a challenge. Herein, we have prepared a novel amino-functional ionic liquid polymer adsorbent (NH_2_-IL-PS) for the extraction of naproxen sodium (NPS) from aqueous environments. It was found that the NH_2_-IL-PS exhibits a high adsorption capacity of 456.6 mg/g for NPS and maintains high extraction efficiency over a wide pH range of 4 to 9 at room temperature. Notably, even when the concentration of NPS was lower than 5 ppb, the extraction efficiency still exceeded 90.0%, with the enrichment factor reaching up to 600.0. More importantly, the NH_2_-IL-PS adsorbent material can withstand at least 16 consecutive adsorption cycles while maintaining an extraction efficiency of over 90.0%. Finally, the NH_2_-IL-PS was successfully applied to extract and determine NPS in seven types of real water samples, with relative recoveries ranging from 90.9 to 96.2%. The study of the adsorption mechanism reveals that electrostatic interactions, ion exchange, π-π stacking, and hydrogen bonding are crucial in the extraction of NPS. This study provides a sustainable strategy for the efficient extraction of NPS.

## 1. Introduction

Naproxen sodium is a type of non-steroidal anti-inflammatory drug [1], commonly used in humans, fisheries, and livestock due to its analgesic, antipyretic, anti-inflammatory, and other effects [2,3,4]. Nonetheless, even long-term consumption of minute quantities of naproxen sodium can still potentially cause heart attacks and strokes, and may exert toxic effects on the lungs [1,5]. Therefore, it is crucial for both human health and environment protection to select an accurate and effective method for determining the presence of naproxen sodium in environmental water.

Determining naproxen sodium directly is challenging due to its very low content in environmental samples. Therefore, samples containing naproxen sodium must undergo separation and enrichment processes prior to determination to meet the method’s requirements. Currently, there are numerous sample pretreatment techniques for naproxen sodium, including liquid–liquid dispersion micro-extraction [6], two-aqueous phase extraction [1], novel stir bar adsorption extraction [7], and solid phase extraction [8,9]. Solid phase extraction has garnered widespread attention due to its advantages of low preparation cost, good adsorption effect, and strong operability [10,11]. For example, Onal et al. [12] examined the adsorption capacity of modified waste apricot activated carbon for naproxen sodium, which was found to be 106.4 mg/g. Guo et al. [8] investigated the efficient extraction ability of dipyridyl organosilica nanosheets for naproxen sodium, an emerging micro-pollutant. Shin et al. [1] explored the adsorption mechanism of seven active pharmaceutical ingredients by comparing the original biochar extracted from waste coffee grounds and the biochar activated by NaOH. Although these works possess their own characteristics, however, the regeneration capacity of biochar adsorbents is limited, the preparation of nanomaterials is complex, and they are prone to causing secondary pollution, with their adsorption capacity being relatively low. Therefore, it is worthwhile to design solid phase extractants with strong adsorption capacity, good adsorption selectivity, simple preparation, and easy regeneration for sample pretreatment research.

Ionic Liquids (ILs) are a class of liquid salt compounds composed entirely of cations and anions, which typically remain liquid at or near room temperature (generally referring to a melting point below 100 °C), which have been successfully applied in many fields, including organic synthesis, material preparation, and separation analysis, due to their low vapor pressure, strong solubility, and designable structure [13]. However, when using ionic liquid alone, there are still several disadvantages, such as a large amount required [14], high cost, ease of loss, and difficulty of recovery [15,16,17]. To address these issues, solid-loaded ionic liquid materials have become a research hotspot. Zhu et al. [18] grafted 1-butyl-3-vinyl imidazolium bromide onto the surface of silica to prepare a simple and feasible ionic liquid functionalized polymer (IL-P), which was used for the separation and enrichment of four trace phenolic residues in complex wastewater and soil samples. Cheng et al. [19] synthesized hydroxyl-functionalized imidazole ionic liquid adsorbents and successfully applied them to remove sodium cefthiophene from water. Fan et al. [20] prepared polystyrene-supported imidazole ionic liquid materials by the chemical medium method and investigated their adsorption performance for sodium sulfadiazine. In this work, we have prepared amino-functionalized polymer adsorption material (NH_2_-IL-PS). The material not only retains the efficient extraction performance and anti-interference capability of both ionic liquid and amino-functional group, but also effectively resolves the challenges of difficult phase separation and loss during liquid–liquid extraction. Additionally, it provides the benefits of a stable and renewable solid carrier. The results indicate that NH_2_-IL-PS is a highly promising material for the extraction of naproxen sodium (NPS) from aqueous environments. The established separation and analysis method provides a new strategy for green pretreatment of water samples and NPS detection.

## 2. Results and Discussion

### 2.1. Characterization of NH_2_-IL-PS

**Figure 1:**
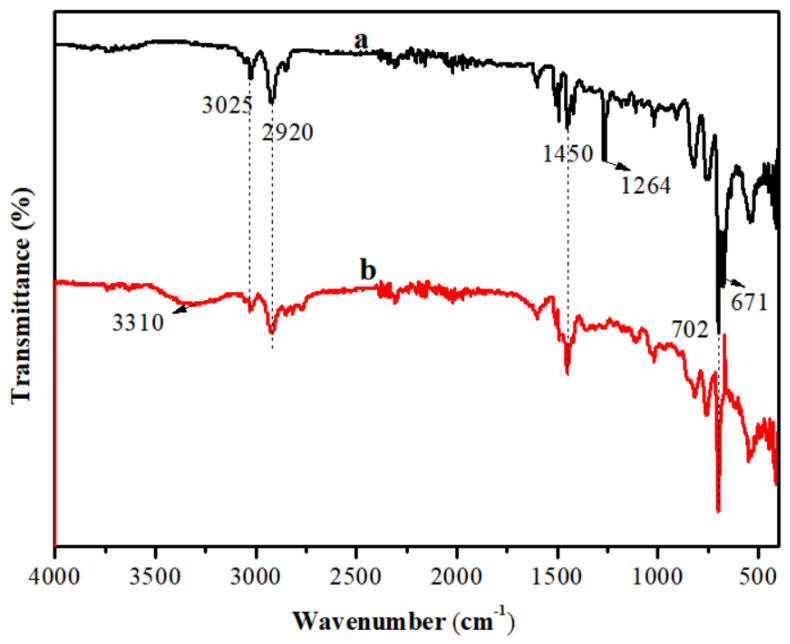
FT-IR spectra of PS-CH_2_Cl (**a**) and NH_2_-IL-PS (**b**).

To demonstrate the successful preparation of NH_2_-IL-PS, the FT-IR spectrum was measured and is shown in Figure 1. It was clearly observed that two distinct peaks at 1260 cm^−1^ and 671 cm^−1^, which were ascribed to the stretching and bending vibration of C-Cl of PS-CH_2_Cl [14], disappeared in NH_2_-IL-PS. However, the emergence of a new peak at 3310 cm^−1^ (associated with N-H stretching vibration) clearly demonstrates that an N-H bond of *N*,*N*-dimethyl ethylenediamine was successfully immobilized in the NH_2_-IL-PS [21,22]. Furthermore, the three characteristic peaks at 3025 cm^−1^, 2920 cm^−1^, and 1450 cm^−1^, which correspond to the C-H stretching vibration of benzene ring, the C-H stretching vibration of methylene, and the C-C skeleton vibration of benzene ring, remained virtually changed [23] before and after the carrier was immobilized. This suggests that the *N*,*N*-dimethyl ethylenediamine group was successfully immobilized on the carrier, and the basic skeleton structure of the carrier remained undamaged throughout the immobilization process.

**Figure 2:**
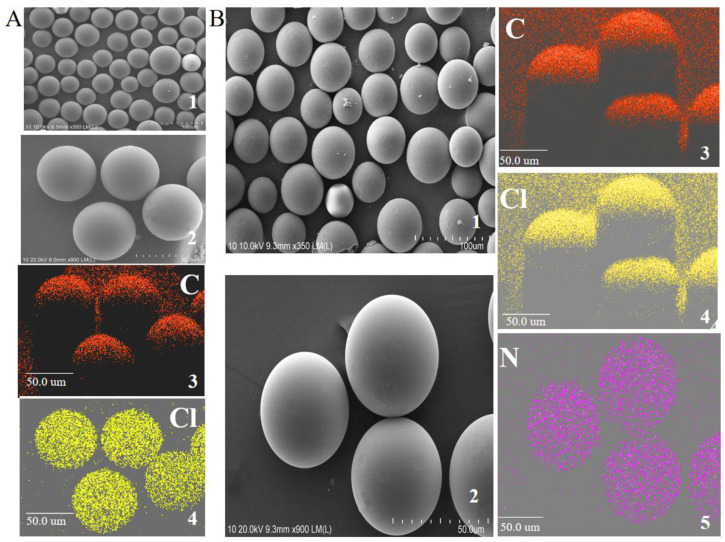
SEM images of PS-CH_2_Cl (**A1**,**A2**) and NH_2_-IL-PS (**B1**,**B2**), EDS mapping of PS-CH_2_Cl (**A3**,**A4**) and NH_2_-IL-PS (**B3**–**B5**).

The morphology of PS-CH_2_Cl and NH_2_-IL-PS were examined using SEM. As depicted in Figure 2(A1,A2,B1,B2), it was observed that both PS-CH_2_Cl and NH_2_-IL-PS exhibited a uniform globular morphology with particle sizes ranging from 90 to 120 μm. However, nitrogen elements were prominently present in NH_2_-IL-PS (Figure 2(B3–B5)), whereas they were absent in PS-CH_2_Cl, indicating that *N*,*N*-dimethyl ethylenediamine was immobilized onto the surface of PS-CH_2_Cl, which is consistent with the analyses of FT-IR.

The thermal stability of NH_2_-IL-PS was reflected by thermogravimetric analysis (TGA). As shown in Figure S1, there was almost no weight loss of the PS-CH_2_Cl in the range of 20~300 °C. However, in the temperature range from 100~200 °C, there was a slight weight loss of NH_2_-IL-PS, which may be attributed to the hydrophilic structure of the *N*,*N*-dimethyl ethylenediamine that promotes NH_2_-IL-PS adsorption of a small amount of water in air. This indicates that NH_2_-IL-PS has excellent thermal stability up to 200 °C. Once the temperature surpasses 300 °C, the PS-CH_2_Cl experiences a weight loss of nearly 75%, primarily due to the breakdown of the carbon–carbon skeleton, the cleavage of carbon-chlorine bonds within functional groups such as methyl chloride, and the condensation of certain aromatic rings into carbonaceous residues [24]. The NH_2_-IL-PS undergoes a weight reduction of nearly 90% between 200 and 800 °C. In addition to the aforementioned reasons, this weight loss may also be attributed to the decomposition of quaternary ammonium salts [25]. Therefore, the amount of *N*,*N*-dimethyl ethylenediamine immobilized on the surface of the carrier was calculated to be close to 15%.

### 2.2. Adsorption Performance of Material

#### 2.2.1. Effects of pH Value

**Figure 3:**
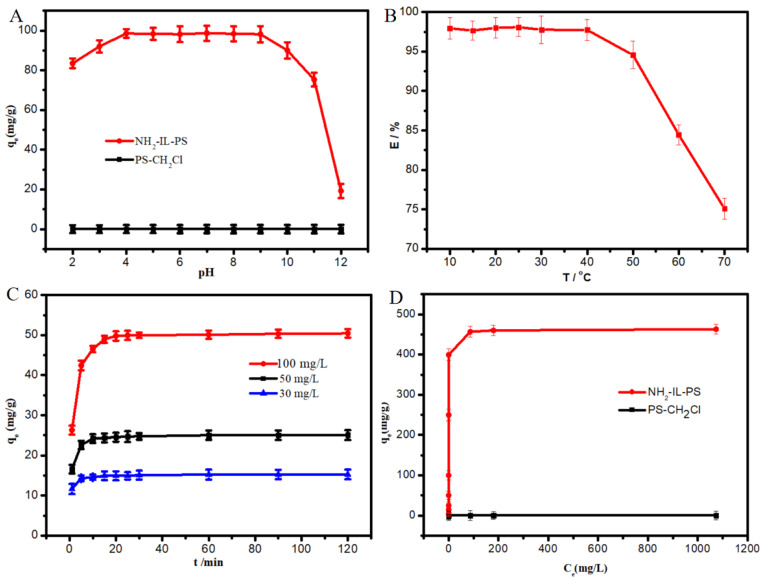
Effect of initial pH (**A**), temperature (**B**), adsorption capacity at different initial concentrations (**C**), and adsorption isotherm (**D**) on the extraction of NPS by the NH_2_-IL-PS. Experimental conditions (**A**): NH_2_-IL-PS: 5 mg; NPS: 100 mg/L, 5 mL; (**B**): NH_2_-IL-PS: 5 mg; NPS: 100 mg/L, 5 mL; (**C**): NH_2_-IL-PS: 10 mg; NPS: 5 mL; (**D**): NH_2_-IL-PS: 5 mg; NPS: 5 mL.

The pH value significantly affects the surface charge of NH_2_-IL-PS and the existing form of NPS. From Figure 3A, it is evident that PS-CH_2_Cl does not adsorb NPS. In contrast, NH_2_-IL-PS demonstrates excellent adsorption performance towards NPS. The ionic fractions of NPS are displayed in Table S1. Given that the pKa of NPS is 4.84 [7], NPS predominantly exists in anionic form when the pH is greater than the pKa. This is advantageous for enhancing the adsorption performance of the adsorbent, as electrostatic interactions play a key role between NPS and NH_2_-IL-PS. However, when the pH is between 9 and 12, the adsorption capacity decreases sharply. The possible reason for this may be competition between OH^−^ and naproxen sodium anion, which directly affects the adsorption of NPS. Overall, NH_2_-IL-PS can effectively adsorb NPS over a wide pH range (4–9), and the subsequent tests were operated at a natural pH value (pH ≈ 6).

#### 2.2.2. Effects of Temperature

The influence of temperature on the adsorption efficiency of NH_2_-IL-PS for NPS was investigated. It is clearly seen from Figure 3B that the temperature from 10 °C to 50 °C has little effect on the adsorption efficiency. However, at temperatures above 50 °C, the extraction efficiency of NH_2_-IL-PS drops dramatically. Subsequent experiments were conducted at room temperature.

#### 2.2.3. Influence of the Equilibrium Time and Analysis of Adsorption Kinetics

To comprehensively investigate the extraction efficiency of the adsorbent for target pollutants in water, the adsorption capacity of NPS at various initial concentrations over different time periods was studied. As depicted in Figure 3C, the equilibrium time was progressively extended as the initial concentration of NPS increased. Nonetheless, overall, the adsorption of the material onto the target pollutant can be achieved within 20 min across various concentrations, which greatly benefits practical applications. To better investigate the adsorption of NPS on NH_2_-IL-PS, the experimental data were fitted using adsorption kinetics (the kinetic equations are shown in Table S2). The results are displayed in Table S3, where the correlation coefficient of pseudo-second-order model (R^2^ = 0.999) is relatively high. Additionally, the theoretical adsorption capacities calculated by the model (15.31 mg/g, 25.21 mg/g, 50.97 mg/g) are very close to the experimental values shown in Figure 3C (15.26 mg/g, 25.06 mg/g, 50.43 mg/g), indicating that the adsorption of NH_2_-IL-PS on NPS conforms to the pseudo-second-order kinetic model.

#### 2.2.4. Adsorption Capacity

To better study the adsorption performance of NH_2_-IL-PS, we investigated its adsorption capacity. From Figure 3D, it is evident that the carrier PS-CH_2_Cl barely adsorbed NPS at various concentrations, whereas the NH_2_-IL-PS demonstrated excellent adsorption properties for NPS. Within the range of 1000 to 2000 mg/L, the adsorption capacity appeared to plateau, suggesting that adsorption had reached saturation. To further investigate the interaction between NPS and NH_2_-IL-PS, the data were fitted by two isotherm models (Table S4), and the results were depicted in Table S5. It was obvious to see that the data were best fitted to the Langmuir model (chi-square value (χ^2^) is 0.024 and root mean square errors (RMSE) is 0.12). Furthermore, the maximum equilibrium adsorption capacity (471.8 mg/g) calculated by this model is very close to the experimental value (456.6 mg/g). All these indicate that the adsorption of NPS by NH_2_-IL-PS is characterized by monolayer homogeneous adsorption, where the stronger adsorption sites initially capture the target pollutant, and, subsequently, the adsorption becomes saturated as these sites are progressively occupied.

### 2.3. Optimization of Experimental Conditions

The conditions of column adsorption experiments, including injection velocity, eluent type and volume, and elution rate, are important factors affecting the extraction performance and recovery of the target analyte. Herein, the conditions for column adsorption experiments were optimized using 30 mL of a 30 mg/mL NPS solution passed through a solid extraction column containing 100 mg of NH_2_-IL-PS. The results are shown in Figure S2, and the details are presented in Section S1 of the Supplementary Material.

### 2.4. Enrichment Efficiency of the NH_2_-IL-PS Adsorbent Material

Naproxen sodium (NPS) is typically present in the environment at ppb levels or lower [9]. To study the separation and enrichment capabilities of NH_2_-IL-PS for trace amounts NPS in water, 1.0 mL 10 mg/L NPS solution was added to a 50–2000 mL volumetric flask and diluted into NPS solution with different concentrations. Subsequently, the enrichment ability was assessed through dynamic experiments. NPS solutions of varying concentrations were passed through NH_2_-IL-PS solid phase extraction columns, and the NPS adsorbed on the columns were eluted using anhydrous ethanol. Under optimal conditions, the solid-phase extraction effect of NPS solutions with varying concentrations is presented in Table 1. Recoveries of NPS exceed 90% within the concentration range of 0.2 to 0.005 mg/L, and the enrichment factor (*EF*) reaches as high as 600.0. The *EF* is calculated using the formula *EF* = *C_f_*/*C_i_* [26], where *C_f_* and *C_i_* represent the concentrations of NPS in the eluent and the initial sample solution, respectively. Such a high enrichment factor is particularly well-suited for the separation, analysis, and determination of trace naproxen sodium residues in environmental samples.

### 2.5. Effect of Coexisting Substances

The actual water samples typically include inorganic and organic ions that may affect the extraction performance of NPS. Therefore, we studied the influence of coexisting substances on the extraction performance of NPS. First, 5 mL of 4.0 × 10^−5^ mol/L NPS was passed through a column containing NH_2_-IL-PS at a flow rate of 5 mL/min, after which the NPS adsorbed on NH_2_-IL-PS was eluted with anhydrous ethanol. The concentration of NPS in the eluent was determined. The recovery rate of NPS was calculated in the absence and presence of interfering substances. It is considered that the concentration of interfering substances does not affect the extraction of NPS by materials when the recovery rate error is within ±5%. Thus, the optimal concentration without interference is determined, and, subsequently, the permissible maximum interference ratio is calculated. It is evident from Table 2 that NH_2_-IL-PS exhibits a strong anti-interference capability during the absorption of NPS. Except for PO_4_^3−^, the interference rates of common anions and cations are all above 1500 times, especially when the concentrations of K^+^, Na^+^, and Cl^−^ are up to 1.0 mol/L, which is 25,000 times the concentration of NPS; the adsorption effect of the prepared SPE material on NPS remained within a 4.7% error range. Although the anti-interference rates of CO_3_^2−^, SO_4_^2−^, and PO_4_^3−^ are lower than those of K^+^, Na^+^, and Cl^−^, the salt content in typical industrial wastewater can reach as high as 12~15% [27], which is a significantly lower concentration than that used in our experiments, suggesting that the material possesses a strong tolerance for high-salt environments and holds a distinctive advantage in the treatment and separation analysis of industrial wastewater containing NPS.

The impact of coexisting organic compounds on the extraction of NPS by NH_2_-IL-PS was examined using the same method. It was observed that the extraction results were minimally affected, even when the concentrations of coexisting organic compounds were 625–25,000 times greater than that of NPS. This suggests that NH_2_-IL-PS is well-suited for the efficient separation and subsequent enrichment of target pollutants in high-salt wastewater.

### 2.6. Sustainability of Naproxen Sodium Wastewater Pretreatment by NH_2_-IL-PS

**Figure 4:**
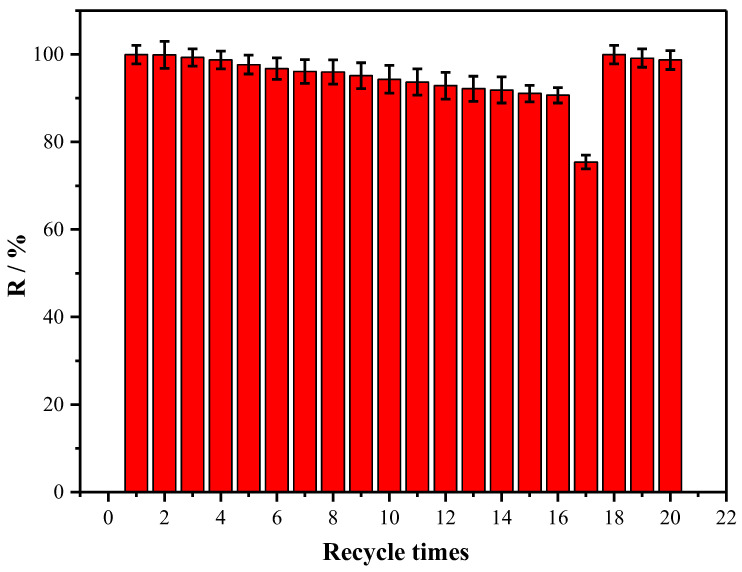
Extraction recovery of NPS by NH_2_-IL-PS during cyclic extraction.

Whether the adsorbent material can be regenerated and recycled is a key factor in investigating the practical value of the adsorbent. In light of this, we have conducted recycling and regeneration experiments on NH_2_-IL-PS. It is evident that the extraction efficiency of NPS was reduced by only about 10% after 16 consecutive adsorptions (Figure 4), yet it experienced a significant decrease of 25% at the 17th consecutive adsorption. The possible reason was that the cumulative adsorption capacity (44.67 mg/g) of the SPE column containing 100 mg of NH_2_-IL-PS was close to the saturated adsorption capacity (45.61 mg/g). After the NPS adsorbed on the NH_2_-IL-PS was eluted off by an eluent, the properties of NH_2_-IL-PS were recovered by the 18th extraction. The adsorption properties of the regenerated NH_2_-IL-PS were almost indistinguishable from those of the original NH_2_-IL-PS and could still be continuously adsorbed until saturation was reached. This not only significantly reduces the amount of eluent required but also shortens the time, which is greatly beneficial for industrial applications.

Additionally, the eluent containing NPS can be recycled through simple heating and distillation, with NPS also being recovered. As evidenced by the infrared spectrum in Figure S3A, the main functional group structure of the newly prepared and regenerated NH_2_-IL-PS material remains unchanged, indicating that NH_2_-IL-PS possesses good chemical stability and can be reused. The absorption spectra of the original NPS and the recovered NPS remain essentially unchanged (Figure S3B), indicating that the recovered drug is relatively pure and no chemical reactions have occurred to form other substances. In conclusion, NPS can be effectively recovered, which can reduce drug residue pollution and facilitate drug recycling. Simultaneously, the NH_2_-IL-PS can be regenerated, the eluent anhydrous ethanol can be recycled, and the NPS in the eluent can be directly determined through simple spectrophotometry, which is easy to operate and does not necessitate the use of HPLC and other large, expensive equipment. Notably, there is essentially no waste generated throughout the entire treatment process. This sustainable recycling sample pretreatment process holds potential practical value in conserving resources, fostering economic development, and protecting the environment.

### 2.7. The Applications of Actual Water Samples

To evaluate the performance of NH_2_-IL-PS in extracting target pollutants in practical applications, the separation and enrichment effects of NH_2_-IL-PS on NPS in seven types of real water samples were investigated, with the results presented in Table 3. The extraction performance of NH_2_-IL-PS for NPS in real water samples diminished with the growing complexity of the water matrix, potentially due to matrix effects. Nonetheless, overall, the material demonstrated robust anti-interference capabilities. The recoveries of NPS in the seven water samples exceeded 90%, suggesting that NH_2_-IL-PS effectively separates and enriches NPS in actual samples.

### 2.8. Comparison with Other Literature Reports and Commercially Available Adsorbents

To further investigate the adsorption advantages of NH_2_-IL-PS in this study, we compared the synthesized material with literature reports and commercial adsorbents. As shown in Table 4, compared to some previously reported adsorbents, NH_2_-IL-PS exhibited significant advantages in terms of adsorption capacity, equilibrium time, and recycling number. Moreover, the adsorption capacity of NH_2_-IL-PS is 2.5–16.9 times greater than that of other commercially available adsorbents, and the recovery rate is 4.9–131.6 times higher than theirs (Table S6). All these results suggest that the prepared NH_2_-IL-PS not only has a large adsorption capacity but is also easy to recycle. It is a type of adsorbent that can effectively separate and concentrate NPS from polluted water.

### 2.9. Analysis of Adsorption Mechanism

It has been reported that hydrogen bonding and π-π stacking play a very important role in the adsorption of NPS [1,8]. To verify these speculations, IR spectra before and after NH_2_-IL-PS adsorption of NPS are the most favorable evidence. The N-H stretching vibration of NH_2_-IL-PS at 3310 cm^−1^ [18] (Figure S4a) and the C=O stretching vibration of NPS at 1572 cm^−1^ [31,32,33] (Figure S4c) both weakened and shifted to 3300 cm^−1^ and 1583 cm^−1^, respectively, after the adsorption of NPS (Figure S4b). This indicates that there may be a hydrogen bonding interaction between the N-H bond on NH_2_-IL-PS and the C=O on NPS. Additionally, the peak of the C-C benzene ring skeleton vibration for NH_2_-IL-PS at 1452 cm^−1^ and NPS at 1478 cm^−1^ shifted to 1457 cm^−1^ after NH_2_-IL-PS adsorbed NPS, indicating that π-π stacking may exist between the material NH_2_-IL-PS and the target pollutant NPS [21,34].

Additionally, considering the effects of initial pH discussed previously, electrostatic interactions may occur during the adsorption process. Concurrently, from the discussion of the anti-interference ability of the material experiment, it is evident that the greater the charge of the ion, the more significant the interference on the adsorption performance of NH_2_-IL-PS. Specifically, the interference from divalent ions is greater than that from univalent ions, indicating that electrostatic effects play a crucial role in the adsorption process.

**Figure 5:**
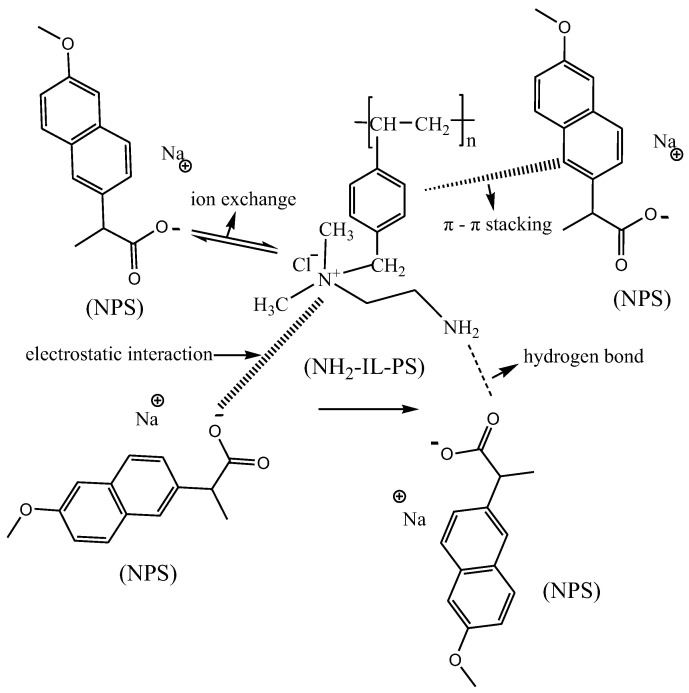
The possible adsorption mechanisms of NPS on NH_2_-IL-PS.

It is also speculated that ion exchange may occur during the adsorption process of NH_2_-IL-PS and naproxen sodium. To verify this hypothesis, a drop of 0.1 mol/L HNO_3_ solution and a drop of 0.5 mol/L AgNO_3_ solution were added to the NPS solution, the supernatant of NH_2_-IL-PS material soaked in deionized water, and the supernatant of NH_2_-IL-PS adsorbed NPS, respectively. As depicted in Figure S5, it was observed that only white precipitation appeared in the supernatant after NH_2_-IL-PS adsorption of NPS, confirming our hypothesis that ion exchange might occur during the adsorption process. These findings indicate that the synergistic effects of hydrogen bonding, π-π stacking, ion exchange, and electrostatic interaction are crucial factors contributing to the material’s effective adsorption of target pollutants. The possible adsorption mechanism is shown in Figure 5.

## 3. Experimental Section

### 3.1. Reagents and Materials

Chloromethyl polystyrene resin (PS-CH_2_Cl, 200–400 mesh, 1% DVB, 3.5 mmol of Cl/g) was obtained from Tianjin Hecheng Co., Ltd. (Tianjin, China). N-methyl-2-pyrrolidone (98%), *N*,*N*-dimethyl ethylenediamine (98%), and naproxen sodium (99%) were obtained from Bai Lingwei Co., Ltd. (Beijing, China).

### 3.2. Preparation of the Amin- Functional Ionic Liquid Polymer

**Figure 6:**
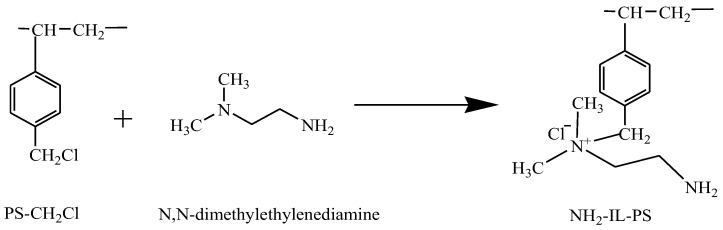
Preparation of NH_2_-IL-PS.

The amino-functional ionic liquid polymer (NH_2_-IL-PS) was synthesized as follows: First, 0.5 g of PS-CH_2_Cl and 20 mL of N-methyl-2-pyrrolidone were added to a 50 mL volumetric flask and allowed to swell for 12 h. Subsequently, 10 mL of 2-dimethylethanolamine was slowly added, and the mixture was reacted at a rate of 500 rpm within an oil bath set at 80 °C for 12 h. The above mixture was transferred to a 50 mL centrifuge tube, washed with deionized water up to 30 mL, vortexed for 5 min, and centrifuged at 8000 rpm for 5 min. The supernatant was discarded, 30 mL of ethanol was added, and the material was washed using the same deionized water washing method. We alternated washing with ethanol and deionized water three times each. (The supernatant was aspirated and its UV–visible absorption spectrum was measured. When the spectrum baseline was essentially consistent with that of ethanol or deionized water scanning, it indicated that the cleaning was thorough.). Finally, the resulting product was vacuum-dried at 60 °C for 12 h. The synthetic reaction is depicted in Figure 6.

### 3.3. Apparatus

The relevant instruments and equipment used in the experiment are displayed in Section S2 of the Supplementary Material.

### 3.4. Adsorption and Determination of Naproxen Sodium

Adsorption experiments of amino-functional ionic liquid polymer (NH_2_-IL-PS) towards naproxen sodium (NPS) were conducted using both batch and column adsorption methods. The effects of pH and temperature on the adsorption performance of the material, as well as the adsorption equilibrium time and adsorption capacity, were investigated by the batch method. The column method was used to study the enrichment factor, selectivity, regenerability, and practical applications of the material, and the further details are provided in the Section S2 of Supplementary Material. The concentration of NPS was determined using UV-Vis spectrophotometry at a wavelength of λmax = 272 nm. The adsorption capacity and recovery rate of NPS were calculated by the equations shown in Section S3 in Supplementary Material.

### 3.5. Pre-Treatment of Real Samples

Seven water samples were collected from various water sources in Xinxiang City (Henan, China), then filtered directly using a 0.22 μm membrane, and stored at 4 °C for subsequent analysis.

## 4. Conclusions

In this work, a novel amino-functional immobilized ionic liquid polymer (NH_2_-IL-PS) was prepared through chemical grafting. The properties of NH_2_-IL-PS, as well as its separation and enrichment effects on NPS in seven environmental water samples, were systematically investigated. The NH_2_-IL-PS exhibited a high adsorption capacity (456.6 mg/g) for NPS from actual water samples over a wide pH range at room temperature. More importantly, the extraction efficiency remained above 90.0% even when the NPS content was lower than 5 ppb, with an enrichment factor reaching up to 600.0. Moreover, NH_2_-IL-PS demonstrated robust anti-interference capabilities. Even at concentrations of K^+^, Na^+^, Cl^−^, and glucose as high as 1.0 mol/L, which is 25,000 times the concentration of NPS, it still exhibited an extremely low impact on the adsorption performance of NPS. This suggests that NH_2_-IL-PS is well-suited for the separation and enrichment of target pollutants in high-salt wastewater. The extraction efficiency of NPS was still above 90% after 16 consecutive adsorptions. The enriched NPS in the eluent can be directly analyzed using simple UV–visible spectrophotometry, a method that is easy to operate and does not necessitate the use of large, expensive instruments. The material can be regenerated, the eluent can be recycled, and the target pollutants can be recovered, aligning with the sustainable principles of green chemistry. This demonstrates that NH_2_-IL-PS is an adsorbent material with significant potential for practical application.

## Figures and Tables

**Table 1:** Extraction performance of NPS with different concentrations by adsorbents.

V_NPS_ (mL)	*C_i_* (μg/mL)	*R* (%)	*C_f_* (μg/mL)	*EF*	RSD (%)
50.0	0.20	98.8	3.30	16.5	1.1
100.0	0.10	97.4	3.25	32.5	1.9
250.0	0.04	95.7	3.19	79.8	1.3
500.0	0.02	93.2	3.11	155.5	2.1
1000.0	0.01	91.2	3.04	304.0	1.5
2000.0	0.005	90.1	3.00	600.0	1.7

Experimental conditions: m_NH2-IL-PS_ = 100 mg, pH ≈ 6, room temperature.

**Table 2:** The permissible interference ratio of common inorganic ions and organics.

Coexisting Substances	Permit Ratio	Concentration (mol/L)	RSD (%)
K^+^	25,000	1.0	1.7
Na^+^	25,000	1.0	2.7
Mg^2+^	10,000	0.4	1.5
Ca^2+^	10,000	0.4	1.1
Cl^−^	25,000	1.0	2.2
CO_3_^2−^	1500	6.0 × 10^−2^	1.3
SO_4_^2−^	1500	6.0 × 10^−2^	1.3
PO_4_^3−^	1000	4.0 × 10^−2^	1.6
starch	1500	6.0 × 10^−2^	2.7
glucose	25,000	1.0	2.5
Sucrose	25,000	1.0	3.9
Humic acid	2500	0.1	4.7
SDBS	625	2.5 × 10^−2^	4.2

SDBS: sodium dodecyl benzene sulfonate; experimental conditions: m_NH2-IL-PS_ = 100 mg, C_NPS_ = 4.0 × 10^−5^ mol/L, pH ≈ 6, room temperature.

**Table 3:** The recovery of naproxen sodium from real water samples.

Sample	Detected (mg/L)	Added Values (mg/L)	R (%)	RSD (%)
Tap water	N D	10.0	96.2	1.2
Rain water	N D	10.0	95.3	2.1
River water	N D	10.0	95.6	1.8
Lake water	N D	10.0	95.5	1.7
The Yellow River water	N D	10.0	95.2	1.9
Sewage effluents	N D	10.0	91.8	2.2
Domestic sewage	N D	10.0	90.9	1.5

Experimental conditions: C_NPS_ = 10 mg/L, t = 30 min, T = 20 °C.

**Table 4:** The comparison of extraction performance of different adsorbents to naproxen sodium.

Adsorbent	q_e_ (mg/g)	t_eq_ (min)	RN (Times)	Ref.
Biochars	48.5	720	-	[1]
MIL-101(Cr)@GA	333.3	30	9	[28]
Dipyridyl-based organo-silica nanosheets	260.6	20	3	[8]
Activated carbon from waste apricot	106.4	30		[12]
Fe_3_O_4_–FeBTC -MOF	69.4	240	3	[29]
Copper nano-adsorbent	33.9	-	-	[30]
Activated carbon from water hyacinth	39.5	480	-	[9]
NH_2_-IL-PS	456.6	20	16	This work

q_e_: adsorption capacity; t_eq_: equilibrium time; RN: recycling number.

## Data Availability

Data is contained within the article or Supplementary Material.

## Supplementary Materials

The following supporting information can be downloaded at: https://www.mdpi.com/article/10.3390/molecules30112404/s1, Section S1. Optimization of experimental conditions for column adsorption experiments; Section S2. The relevant instruments and equipment used in the experiment; Section S3. The adsorption capacity and recovery rate of NPS were calculated by the following equations; Table S1. The ionization degree of NPS at different pH values; Table S2. The kinetic models and their equations; Table S3. Kinetic parameters for the adsorption of NPS by NH_2_-IL-PS; Table S4. The isotherm model and its equations; Table S5. The ionization degree of NPS at different pH values; Table S6. Comparison for the extraction efficiency and recovery of NPS by different adsorbents; Figure S1. The TGA curves of PS-CH_2_Cl (a) and NH_2_-IL-PS (b); Figure S2. Optimization of experimental conditions for dynamic adsorption of NPS by NH_2_-IL-PS (A: Extraction efficiency of NPS at different injection flow speed; B: Recovery rate of NPS in different eluents; C: Recovery of NPS at different elution volumes; D: Recovery rate of NPS at different elution flow speed); Figure S3. (A) FT-IR spectra of the fresh (a) and the regenerated (b) NH_2_-IL-PS; (B) UV-visible absorption spectra of naproxen sodium (a) and the recovered naproxen sodium (b); Figure S4. FT-IR spectra of the NH_2_-IL-PS (a); NH_2_-IL-PS-NPS (b); NPS (c); Figure S5. (a) NPS+AgNO_3_ solution; (b) Supernatant of NH_2_-IL-PS+AgNO_3_ solution soaked in deionized water; (c) Supernatant+AgNO_3_ solution after adsorption of NPS by NH_2_-IL-PS.
