# Supplementary material for: A Novel Recyclable Strategy for Extraction of Naproxen Sodium from Environmental Water by Amino-Functionalized Immobilized Ionic Liquid Polymers
Source: Molecules. 2025 May 30;30(11):2404. doi: 10.3390/molecules30112404 (PMC12155640; doi:10.3390/molecules30112404)
Supplement: Supplementary file 1 [file molecules-30-02404-s001.zip › molecules-3616894-supplementary.pdf]

Supplementary material

A Novel Recyclable Strategy for Extraction of Naproxen Sodium from Environmental Water by Amino-functionalized Immobilized Ionic Liquid Polymers

Hongrui Yang[a,b], Ayiguli Maimaiti[a], Wei Liu[a], Wenye Deng[a], Xiaoping Fu[a], Jing Fan[b*]

[a.]Xinjiang Key Laboratory of New Energy Materials and Green Chemical Engineering, College of Chemical and Environmental Engineering, Xinjiang Institute of Engineering, Urumqi, 830023, China

[b.]School of Environment, Key Laboratory of Yellow River and Huai River Water Environment and Pollution Control, Ministry of Education, Henan Key Laboratory for Environmental Pollution Control, Henan Normal University], Xinxiang, Henan 453007, China

[*] Correspondence: fanjing@htu.cn (J.F.)

Corresponding author: Jing Fan

Address correspondence at: School of Environment, Henan Normal University, Xinxiang, Henan 453007, P. R. China

Tel: +86-373-3325719

Fax: +86-373-3325719

E-mail: fanjing@htu.edu.cn

Table of contents:

1. **Section S1**. Optimization of experimental conditions for column adsorption experiments
2. **Section S2**. The relevant instruments and equipment used in the experiment
3. **Section S3**. The adsorption capacity and recovery rate of NPS were calculated by the following equations.
4. **Table S1.** The ionization degree of NPS at different pH values
5. **Table S2.** The kinetic models and their equations
6. **Table S3.** Kinetic parameters for the adsorption of NPS by NH_2-IL-PS
7. **Table S4.** The isotherm model and its equations
8. **Table S5.** The related parameters of various adsorption isotherm models
9. **Table S6.** Comparison for the extraction efficiency and recovery of NPS by different adsorbents
10. **Fig. S1.** The TGA curves of PS-CH_2Cl (a) and NH_2-IL-PS (b)
11. **Fig. S2.** Optimization of experimental conditions for dynamic adsorption of NPS by NH_2-IL-PS (A: Extraction efficiency of NPS at different injection flow rate; B: Recovery rate of NPS in different eluents; C: Recovery of NPS at different elution volumes; D: Recovery rate of NPS at different elution flow rate)
12. **Fig. S3.** (A) FT-IR spectra of the fresh (a) and the regenerated (b) NH_2-IL-PS; (B) UV-visible absorption spectra of naproxen sodium (a) and the recovered naproxen sodium (b)
13. **Fig. S4.** FT-IR spectra of the NH_2-IL-PS (a); NH_2-IL-PS-NPS (b); NPS (c)
14. **Fig. S5.** (a) NPS+$AgNO_3$ solution; (b) Supernatant of NH_2-IL-PS+$AgNO_3$ solution soaked in deionized water; (c) Supernatant+$AgNO_3$ solution after adsorption of NPS by NH_2-IL-PS

Section S1. Optimization of experimental conditions for column adsorption experiments

To obtain good extraction efficiency, the influence of injection velocity on extraction efficiency was studied. As shown in Fig. S3 (A), when the injection flow rate was 1-5.5 mL/min, the extraction efficiency of NH_2-IL-PS on NPS was the highest and stable. When the injection flow rate was greater than 5.5 mL/min, the extraction efficiency gradually decreased, therefore, 5 mL/min was selected as the injection flow rate in subsequent experiments.

The recovery rate of NPS by different types of eluents were investigated, and the results were shown in Fig. S3 (B). Among 17 eluents with different types and mixing ratios, absolute ethanol had the highest recovery rate. The possible reason was that NPS had a strong solubility in absolute ethanol, so that the NPS adsorbed on NH_2-IL-PS was easily extracted, and absolute ethanol was selected as eluent in subsequent experiments.

In addition, the dosage of eluent was also researched. It was seen in Fig. S3 (C), when the volume of eluent was between 1 and 2.5 mL, the recovery rate of NPS gradually increased, and when the volume of eluent was between 2.5 and 5.0 mL, the recovery rate reached the maximum and was stable. The possible reason may be that NPS adsorbed on the material was eluted completely with 2.5 mL eluent, therefore, the recovery rate of NPS did not increase with the eluent volume in the range of 2.5-5.0, and thus the volume of eluent was selected as 3 mL.

The elution flow speed affects the contact time and elution effect between the eluent and the target pollutant. The recovery rate of NPS was investigated at different elution flow rate. As shown in Fig. S3 (D), the recovery rate of NPS was the highest and stable when the elution flow rate was 0.1-0.4 mL/min. The possible reason is that the higher the elution velocity, the shorter the contact time between the eluent and the target pollutant, which makes it difficult to elute the target pollutant, and thus affects the recovery rate of the pollutant. Considering the recovery rate and elution time, the elution flow rate was chosen to be 0.3 mL/min in the subsequent experiments.

Section S2. The relevant instruments and equipment used in the experiment

A UV–Vis spectrophotometer (TU-1810, Beijing, China) was used for UV-Vis analysis of naproxen sodium. The morphology and thermal stability of the materials were characterized by a Hitachi SU8010 scanning electron microscopy (Tokyo, Japan) and a Netzsch STA449C thermal analyzer (Selb, Germany), respectively. A FTS-40 fourier-transform infrared spectroscopy (Waltham, USA) was used to identify functional groups and interactions between materials and contaminants. A DC101-C oil bath (Zhengzhou, China) was used as the thermostat bath for the synthesis purpose. SPE-12B solid phase extraction instrument (Tianjin, China) was used for the column adsorption experiments.

Section S3. The adsorption capacity and recovery rate of NPS were calculated by the following equations.

$$q_e = \frac{(C_0 - C_e)V}{m} \tag{1}$$

$$q_t = \frac{(C_0 - C_t)V}{m} \tag{2}$$

$$R\% = \frac{(C_0 - C_e)}{C_0} \tag{3}$$

where q_e and q_t are the equilibrium adsorption capacity and the adsorption capacity at contact time t; C_0 (mg/mL), C_e (mg/mL) and C_t (mg/mL) are the concentrations of naproxen sodium at the beginning, equilibrium and time t, respectively; m (mg) is the mass of the adsorbent, V (mL) is volume of NPS solution, and R% stands for the recovery efficiency.

Table S1. The ionization degree of NPS at different pH values (pKa=4.84)

pH	φ_{ions}	pH	φ_{ions}
2.0	1.445×10^{-3}	8.0	0.999
3.0	1.424×10^{-2}	9.0	0.999
4.0	1.263×10^{-1}	10.0	0.999
5.0	5.911×10^{-1}	11.0	0.999
6.0	0.935	12.0	0.999
7.0	0.993		

$$\varphi_{ions} = \frac{1}{[1+10^{(pKa-pH)}]}$$

Table S2. The kinetic models and their equations

Kinetic model	Equation
pseudo-first-order kinetic model	$\ln(q_e\text{-}q_t)=\ln q_e - k_1 t$..................(1)
pseudo-second-order kinetic model	$\frac{t}{q_t} = \frac{1}{k_2 q_e^2} + \frac{t}{q_e}$ (2)

where q_e (mg/g) and q_t (mg/g) are, respectively, the equilibrium adsorption capacity and the adsorption capacity at time t. $k_1$and k_2 are the constant of pseudo-first-order kinetic model and pseudo-second-order kinetic model.

Table S3. Kinetic parameters for the adsorption of NPS by NH_2-IL-PS

C_0（mg/L）	pseudo-first-order model			pseudo-second-order model		
	k_1	q_e（mg/g）	R^2	k_2	q_e（mg/g）	R^2
30	0.0708	11.69	0.894	0.1483	15.31	0.999
50	0.0812	23.89	0.785	0.0729	25.21	0.999
100	0.0677	67.10	0.600	0.0256	50.97	0.999

Table S4. The isotherm model and its equations

Isotherm model	Equation
Langmuir	$\frac{C_e}{q_e} = \frac{C_e}{q_m} + \frac{1}{q_m K_L}$ (1)
Freundlich	$q_e = K_F \cdot C_e^{1/n}$............(2)

where q_e (mg/g) and q_m (mg/g) are, respectively, the equilibrium adsorption capacity and the maximum adsorption capacity. C_e (mg/L) stands for the equilibrium concentration of NPS, K_L and K_F are the constant of Langmuir and Freundlich, respectively.

Table S5. The related parameters of various adsorption isotherm models

Langmuir model				Freundlich model			
K_L(L/mg)	q_m(mg/g)	χ^2	RMSE	K_F	n	χ^2	RMSE
1.31×10^{-3}	471.8	0.024	0.12	0.496	1.71	0.89	0.67

Table S6. Comparison for the extraction performance and recovery of NPS by different adsorbents

Adsorbent	$C_{initial\ NPS}$=10 μg/mL		
	E（%）	q_e（mg/g）	R（%）
Activated carbon	39.70	3.97	20.03
Activated alumina	12.10	1.21	4.98
Silica gel	14.20	1.42	3.35
Artificial zeolite	5.9	0.59	0.75
Weak-base anion exchange	39.6	3.96	14.51
NH_2-IL-PS（this work）	99.6	9.96	98.7

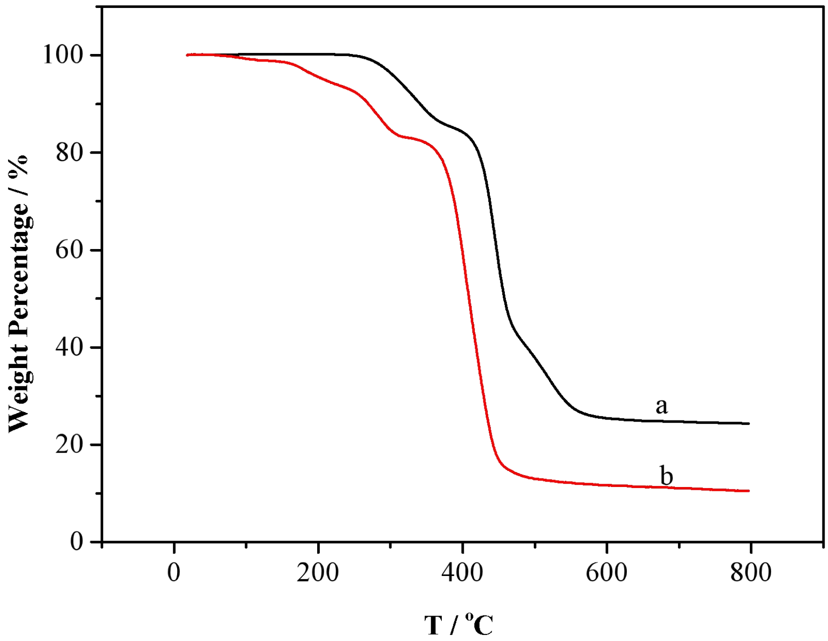

Fig. S1. The TGA curves of PS-CH_2Cl (a) and NH_2-IL-PS (b)

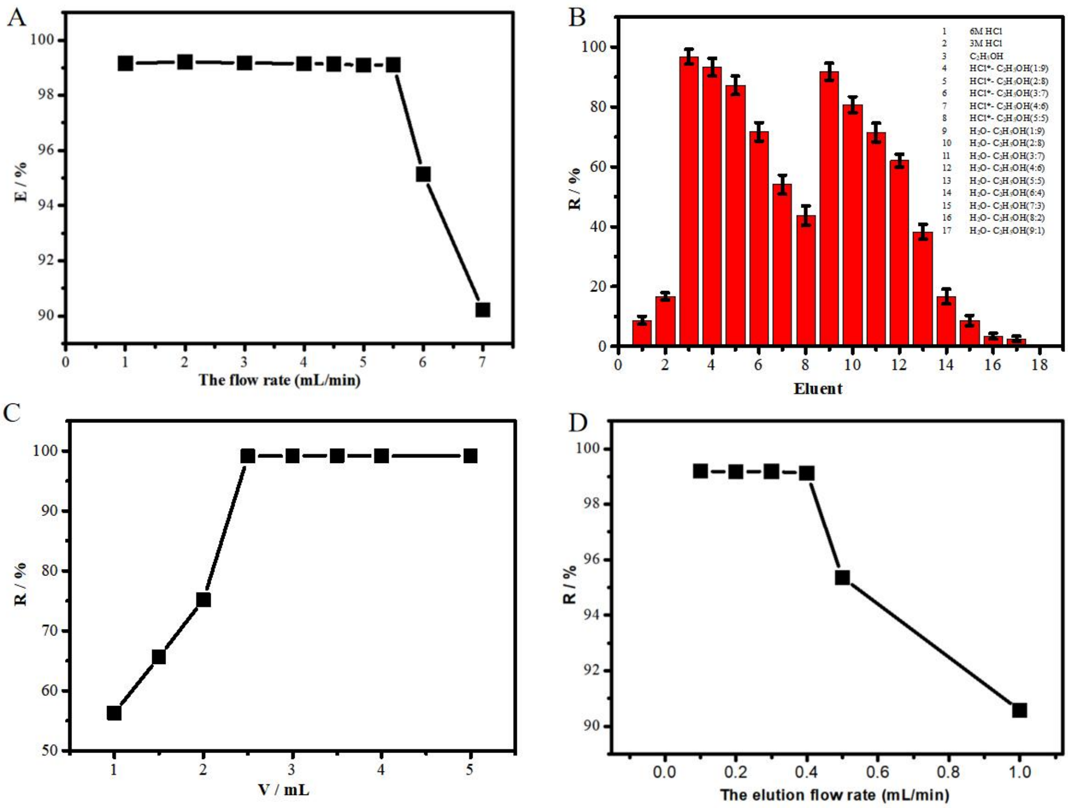

Fig. S2. Optimization of experimental conditions for dynamic adsorption of NPS by NH_2-IL-PS (A: Extraction efficiency of NPS at different injection flow rate; B: Recovery rate of NPS in different eluents; C: Recovery of NPS at different elution volumes; D: Recovery rate of NPS at different elution flow rate)

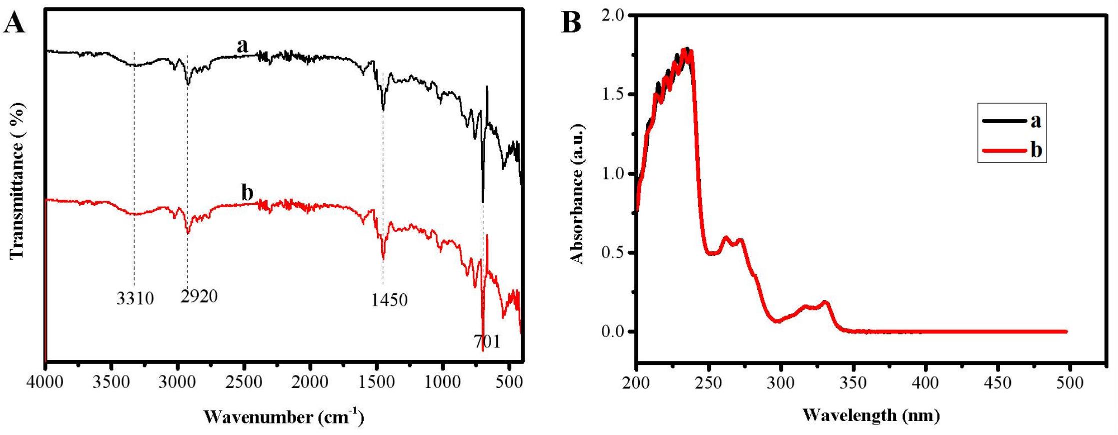

Fig. S3. (A) FT-IR spectra of the fresh (a) and the regenerated (b) NH_2-IL-PS; (B) UV-visible absorption spectra of naproxen sodium (a) and the recovered naproxen sodium (b)

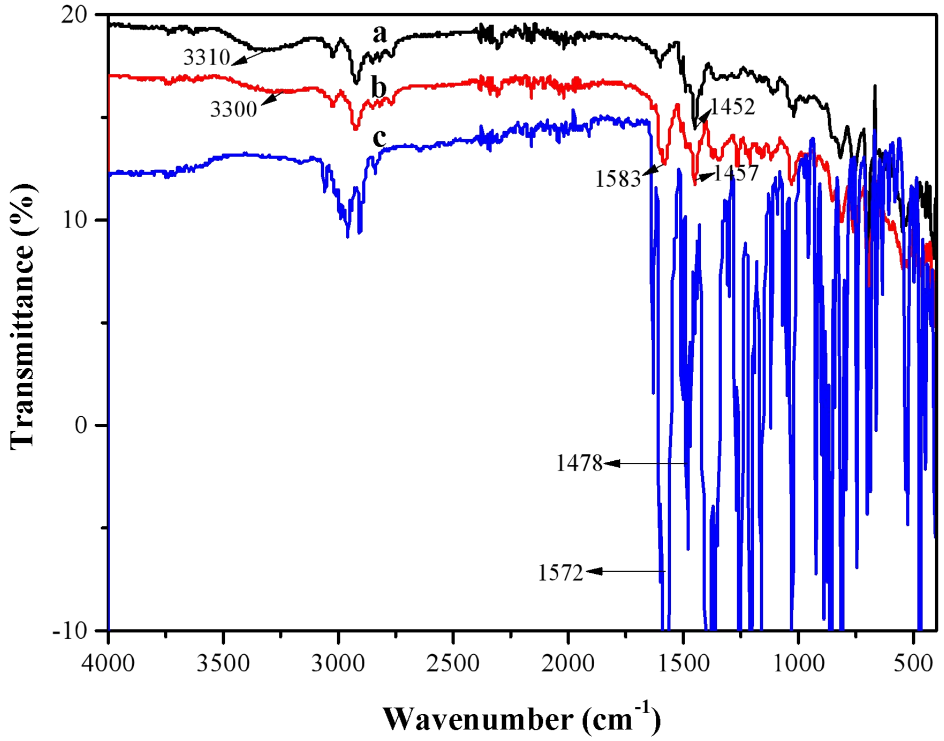

Fig. S4. FT-IR spectra of the NH_2-IL-PS (a); NH_2-IL-PS-NPS (b); NPS (c)

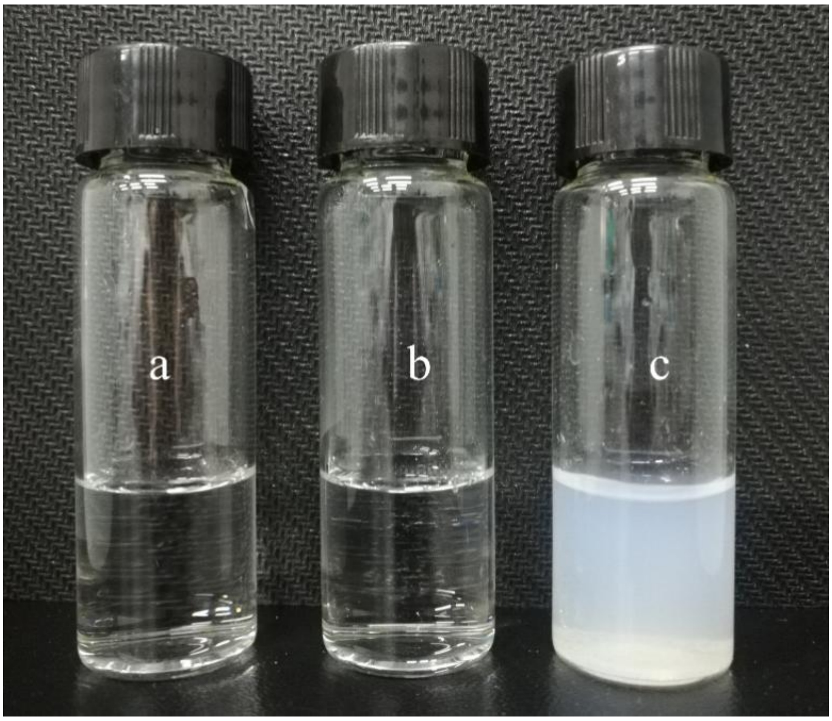

Fig. S5. (a) NPS+$AgNO_3$ solution; (b) Supernatant of NH_2-IL-PS+$AgNO_3$ solution soaked in deionized water; (c) Supernatant+$AgNO_3$ solution after adsorption of NPS by NH_2-IL-PS
